# ﻿Identification of two new species and a new host record of *Distoseptispora* (Distoseptisporaceae, Distoseptisporales, Sordariomycetes) from terrestrial and freshwater habitats in Southern China

**DOI:** 10.3897/mycokeys.102.115452

**Published:** 2024-02-09

**Authors:** Xue-Mei Chen, Xia Tang, Jian Ma, Ning-Guo Liu, Saowaluck Tibpromma, Samantha C. Karunarathna, Yuan-Pin Xiao, Yong-Zhong Lu

**Affiliations:** 1 School of Food and Pharmaceutical Engineering, Guizhou Institute of Technology, Guiyang 550003, China School of Food and Pharmaceutical Engineering, Guizhou Institute of Technology Guiyang China; 2 Center for Yunnan Plateau Biological Resources Protection and Utilization, College of Biological Resource and Food Engineering, Qujing Normal University, Qujing, Yunnan 655011, China Qujing Normal University Qujing China; 3 Engineering and Research Center for Southwest Biopharmaceutical Resource of National Education Ministry of China, Guizhou University, Guiyang, 550025, Guizhou Province, China Guizhou University Guiyang China; 4 Center of Excellence in Fungal Research, Mae Fah Luang University, Chiang Rai, 57100, Thailand Mae Fah Luang University Chiang Rai Thailand; 5 National Institute of Fundamental Studies, Kandy, Sri Lanka National Institute of Fundamental Studies Kandy Sri Lanka

**Keywords:** 2 new taxa, asexual morph, phylogeny, taxonomy

## Abstract

During our investigation of saprophytic fungi in Guizhou and Hainan provinces, China, three hyphomycetes were collected from terrestrial and freshwater habitats. Based on morphological characteristics and phylogenetic analyses of combined ITS, LSU, *tef*1-α, and *rpb*2 sequence data, two new species are introduced: *Distoseptisporahainanensis* and *D.lanceolatispora*. Additionally, one known species, *D.tectonae*, previously unreported from *Edgeworthiachrysantha*, is newly reported. Detailed descriptions, illustrations, and a phylogenetic tree to show the two new species and the new host record of *Distoseptispora* are provided. In addition, a checklist of *Distoseptispora* species with their locations, lifestyles, habitats, and hosts is provided.

## ﻿Introduction

*Distoseptispora* K.D. Hyde, McKenzie & Maharachch. was introduced by Su et al. (2016) with *D.fluminicola* McKenzie, Hong Y. Su, Z.L. Luo & K.D. Hyde, as the type species. Most *Distoseptispora* species are reported as saprophytes, typically found on decaying wood in terrestrial and freshwater habitats (Hyde et al. 2016, 2019; Su et al. 2016; Xia et al. 2017; Yang et al. 2018; Crous et al. 2019; Luo et al. 2019). The initial descriptions of *Distoseptispora* are derived from its asexual morphology (Hyde et al. 2016, 2019, 2020; Su et al. 2016; Yang et al. 2018, 2021; Luo et al. 2019; Sun et al. 2020). The first description of a sexual morph of *Distoseptispora* was described by Yang et al. (2021). Recently, Konta et al. (2023) identified the second sexual species on dead leaves of *Licualaglabra*, and provided detailed explanations, enhancing our understanding of *Distoseptispora* sexual morphology. This sexual morph is characterized by solitary or gregarious, immersed to semi-immersed, subglobose to ellipsoidal, perithecial, dark brown ascomata with a short neck; 8-spored, cylindrical, short pedicellate asci with non-amyloid apical annuli; and fusiform, 0–3-septate, hyaline ascospores with mucilaginous sheaths (Yang et al. 2021; Konta et al. 2023). The asexual morph of *Distoseptispora* was recently expanded upon by Yang et al. (2021), incorporating macronematous, mononematous, solitary or fasciculate conidiophores, blastic, terminal, percurrent, cylindrical conidiogenous cells; and acrogenous, solitary, obclavate, ellipsoidal, obovoid or fusiform, rostrate or not, euseptate, distoseptate or rarely muriform conidia with or without a septal pore and mucilaginous sheath.

*Distoseptispora* has been found on various hosts viz. *Tectona*, *Pandanus*, bamboo, *Clematis*, *Carex*, *Dipterocarpus*, *Licualaglabra*, *Cocosnucifera*, *Phragmitesaustralis*, *Thysanolaenamaxima*, *Platanusorientalis*, and decaying wood and grasses (Shoemaker and White 1985; McKenzie 1995; Hyde et al. 2016, 2019, 2021, 2023; Su et al. 2016; Tibpromma et al. 2018; Crous et al. 2019; Phookamsak et al. 2019; Phukhamsakda et al. 2020, 2022; Sun et al. 2020; Zhai et al. 2022; Afshari et al. 2023; Hu et al. 2023; Konta et al. 2023). Most *Distoseptispora* species have been described in Asia, mainly in China, Thailand, and Malaysia, and only a few have been described in Europe (Shoemaker and White 1985; McKenzie 1995; Phookamsak et al. 2019; Ma et al. 2022; Zhai et al. 2022; Zhang et al. 2022; Konta et al. 2023). *Distoseptispora* comprises 74 accepted species in Index Fungorum (2024), but there is an ambiguity in the taxonomic status of *D.submersa* Z.L. Luo, K.D. Luo et al. (2019) stated that *D.submersa* is phylogenetically closely related to *D.tectonae*, and there are only minor size differences in conidiophores and conidia between *D.tectonae* and *D.submersa*. Dong et al. (2021) synonymized *D.submersa* under *D.tectonae*, thus, *Distoseptispora* comprises 73 accepted saprobic species, of which 44 were from freshwater habitats, 29 from terrestrial habitats, and five from both terrestrial and freshwater environments (Hyde et al. 2016, 2019; Luo et al. 2019; Monkai et al. 2020; Yang et al. 2021; Ma et al. 2022; Zhang et al. 2022; Afshari et al. 2023; Hu et al. 2023; Konta et al. 2023; Liu et al. 2023).

In this study, three fresh hyphomycetous fungal collections were encountered during a microfungal investigation in Hainan and Guizhou provinces. Based on multi-gene phylogeny and morphological comparison, two new species, *Distoseptisporahainanensis* and *D.lanceolatispora* are introduced. In addition, a new host record of *D.tectonae* from *Edgeworthiachrysantha* is also reported.

## ﻿Materials and methods

### ﻿Sample collection, isolation, and morphological study

Fresh specimens were collected from Hainan and Guizhou provinces in China. Fungal colonies were mounted on a slide with distilled water and were observed and examined using a stereomicroscope (SMZ 745, Nikon, Tokyo, Japan). Micro-morphological characteristics were captured with a Nikon EOS 90D digital camera combined with an ECLIPSE Ni-U compound microscope (Nikon, Tokyo, Japan). The sizes of the fungal structures were measured using the Tarosoft (R) Image Frame Work program (IFW 0.97 version), and the photo plates were processed with Adobe Photoshop CC 2019 (Adobe Systems, San Jose, CA, USA).

Single spore isolations were carried out following the methods described in Senanayake et al. (2020). Germinated conidia were transferred to fresh potato dextrose agar (PDA) plates and incubated at 25–27 °C for four weeks. Culture characteristics, including color, shape, and size, were recorded. Herbarium specimens were deposited in the herbarium of the Guizhou Academy of Agriculture Sciences (**GZAAS**), Guiyang, China, and the living cultures were deposited at the Guizhou Culture Collection, China (**GZCC**). Faces of Fungi and Index Fungorum numbers were obtained following the protocols outlined by Jayasiri et al. (2015) and Index Fungorum (2024), respectively.

### ﻿DNA extraction, PCR amplification, and sequencing

Fresh mycelia were scraped from cultures that were incubated at 25–27 °C for 28 days. Fungal genomic DNA was extracted using the Biospin Fungus Genomic DNA Extraction Kit (BioFlux, Shanghai, China), following the manufacturer’s instructions. Four gene regions: internal transcribed spacer (ITS), large subunit ribosomal DNA (LSU), translation elongation factor 1-alpha (*tef*1-α), and RNA polymerase II second largest subunit (*rpb*2) were selected. The primers used in this study for each gene region were as follows: ITS4 and ITS5 for ITS (White et al. 1990), LR0R and LR5 for LSU (Vilgalys and Hester 1990; Cubeta et al. 1991), EF1-983F and EF1-2218R for *tef*1-α (Rehner and Samuels 1994), and *rpb*2 with fRPB2-5F and fRPB2-7cR (Liu et al. 1999).

Polymerase chain reaction (PCR) amplifications were carried out in a 50 µL reaction volume containing 44 μL of 1.1 × T3 Super PCR Mix (TsingKe Biotech, Chongqing, China), 2 µL of DNA template, and 2 µL of each forward and reverse primer. The amplification condition for LSU and ITS consisted of initial denaturation at 94 °C for 3 min, followed by 35 cycles of 45 s at 94 °C, 50 s at 56 °C, and 1 min at 72 °C, and a final extension period of 10 min at 72 °C. The amplification condition for the *tef*1-α gene consisted of initial denaturation at 94 °C for 3 min, followed by 30 cycles of 30 s at 94 °C, 50 s at 56 °C, and 1 min at 72 °C, a final extension period of 10 min at 72 °C. The amplification condition for the *rpb*2 gene consisted of initial denaturation at 95 °C for 5 min, followed by 35 cycles of 15 s at 95 °C, 50 s at 56 °C, and 1 min at 72 °C, a final extension period of 10 min at 72 °C. The quality of PCR amplification products was examined with 1% agarose electrophoresis gels stained with ethidium bromide, and the PCR products were sent to TsingKe Biotech, Chongqing, China for purification and sequencing.

### ﻿Phylogenetic analyses

The raw sequences were initially checked with BioEdit v 7.0.5.3 (Hall 1999). Forward and reverse sequences were assembled using SeqMan v. 7.0.0 (DNASTAR, Madison, WI, USA). Sequence data (LSU, ITS, *tef*1-α, and *rpb*2) for *Distoseptispora* were downloaded from GenBank based on the blast results and recent publications (Table 1). Each individual gene dataset was aligned using the online program MAFFT version 7 with the “auto” option (Hall 1999; Katoh and Standley 2013). These alignments were visually inspected and manually improved in BioEdit v 7.0.5.3. Multi-gene alignments were combined by SequenceMatrix (Vaidya et al. 2011). In this study, phylogenetic analyses were performed using maximum likelihood (ML), maximum parsimony (MP), and Bayesian posterior probability (BYPP) methods. The analyses were based on LSU, ITS, *tef*1-α, and *rpb*2 combined sequence datasets.

**Table 1.** Names, strain numbers, and corresponding GenBank accession numbers of taxa used in this study.

Taxa names	Strain	GenBank Accessions				References
		LSU	ITS	*tef*1-α	*rpb*2	
*Aquapteridosporaaquatica*	MFLUCC 17-2371^T^	MW287767	MW286493	N/A	N/A	Dong et al. (2021)
*Distoseptisporaadscendens*	HKUCC 10820	DQ408561	N/A	N/A	DQ435092	Shenoy et al. (2006)
*D.amniculi*	MFLU 17-2129^T^	MZ868761	MZ868770	N/A	MZ892982	Yang et al. (2021)
*D.appendiculata*	MFLUCC 18-0259^T^	MN163023	MN163009	MN174866	N/A	Luo et al. (2019)
*D.aqualignicola*	KUNCC 21-10729^T^	ON400845	OK341186	OP413480	OP413474	Zhang et al. (2022)
*D.aquamyces*	KUNCC 21-10731^T^	OK341199	OK341187	OP413482	OP413476	Zhang et al. (2022)
*D.aquatica*	MFLUCC 15-0374^T^	KU376268	MF077552	N/A	N/A	Su et al. (2016)
	MFLUCC 18-0646	MK849793	MK828648	N/A	N/A	Luo et al. (2019)
*D.aquisubtropica*	GZCC 22-0075^T^	ON527941	ON527933	ON533677	ON533685	Ma et al. (2022)
*D.atroviridis*	GZCC 20-0511^T^	MZ868763	MZ868772	MZ892978	MZ892984	Yang et al. (2021)
*D.bambusae*	MFLUCC 20-0091^T^	MT232718	MT232713	MT232880	MT232881	Sun et al. (2020)
	MFLUCC 14-0583	MT232717	MT232712	N/A	MT232882	Sun et al. (2020)
*D.bambusicola*	GZCC 21-0667^T^	MZ474872	MZ474873	N/A	N/A	Hyde et al. (2023)
*D.bangkokensis*	MFLUCC 18-0262^T^	MZ518206	MZ518205	N/A	N/A	Shen et al. (2021)
*D.cangshanensis*	MFLUCC 16-0970^T^	MG979761	MG979754	MG988419	N/A	Luo et al. (2018)
*D.caricis*	CPC 36498^T^	MN567632	MN562124	N/A	MN556805	Crous et al. (2019)
	CPC 36442	N/A	MN562125	N/A	MN556806	Crous et al. (2019)
*D.chinensis*	GZCC 21-0665^T^	MZ474867	MZ474871	MZ501609	N/A	Hyde et al. (2021)
*D.clematidis*	MFLUCC 17-2145^T^	MT214617	MT310661	N/A	MT394721	Phukhamsakda et al. (2020)
*D.crassispora*	KUMCC 21-10726^T^	OK341196	OK310698	OP413479	OP413473	Zhang et al. (2022)
*D.curvularia*	KUMCC 21-10725^T^	OK341195	OK310697	OP413478	OP413472	Zhang et al. (2022)
*D.cylindricospora*	DLUCC 1906^T^	OK513523	OK491122	OK524220	N/A	Phukhamsakda et al. (2022)
*D.dehongensis*	KUMCC 18-0090^T^	MK079662	MK085061	MK087659	N/A	Hyde et al. (2019)
*D.dipterocarpi*	MFLUCC 22-0104^T^	OP600052	OP600053	N/A	OP595140	Afshari et al. (2023)
*D.effusa*	GZCC 19-0532^T^	MZ227224	MW133916	N/A	N/A	Yang et al. (2021)
*D.euseptata*	MFLUCC 20-0154^T^	MW081544	MW081539	N/A	MW151860	Li et al. (2021)
	MFLU 20-0568	MW081545	MW081540	MW084994	MW084996	Li et al. (2021)
*D.fasciculata*	KUMCC 19-0081^T^	MW287775	MW286501	MW396656	N/A	Dong et al. (2021)
*D.fluminicola*	MFLUCC 15-0417^T^	KU376270	MF077553	N/A	N/A	Su et al. (2016)
*D.fusiformis*	GZCC 20-0512^T^	MZ868764	MZ868773	MZ892979	MZ892985	Yang et al. (2021)
*D.gasaensis*	HJAUP C2034^T^	OQ942891	OQ942896	OQ944455	N/A	Hu et al. (2023)
*D.guanshanensis*	HJAUP C1063^T^	OQ942898	OQ942894	OQ944452	OQ944458	Hu et al. (2023)
*D.guizhouensis*	GZCC 21-0666^T^	MZ474869	MZ474868	MZ501610	MZ501611	Hyde et al. (2021)
*D.guttulata*	MFLUCC 16-0183^T^	MF077554	MF077543	MF135651	N/A	Yang et al. (2018)
	DLUCC B43	MN163016	MN163011	N/A	N/A	Luo et al. (2019)
***D.hainanensis***	**GZCC 22**-**2047^T^**	**OR438894**	**OR427328**	**OR449122**	**OR449119**	**This study**
*D.hyalina*	MFLUCC 17-2128^T^	MZ868760	MZ868769	MZ892976	MZ892981	Yang et al. (2021)
*D.hydei*	MFLUCC 20-0481^T^	MT742830	MT734661	N/A	MT767128	Monkai et al. (2020)
*D.jinghongensis*	HJAUP C2120^T^	OQ942893	OQ942897	OQ944456	N/A	Hu et al. (2023)
*D.lancangjiangensis*	KUN-HKAS 112712^T^	MW879522	MW723055	N/A	MW882260	Shen et al. (2021)
***D.lanceolatispora***	**GZCC 22**-**2045^T^**	**OR43BB95**	**OR427329**	**OR449123**	**OR449120**	**This study**
*D.leonensis*	HKUCC 10822	DQ408566	N/A	N/A	DQ435089	Shenoy et al. (2006)
*D.licualae*	MFLUCC 14-1163A^T^	ON650675	ON650686	ON734007	N/A	Konta et al. (2023)
	MFLUCC 14-1163B^T^	ON650676	ON650687	ON734008	N/A	Konta et al. (2023)
*D.lignicola*	MFLUCC 18-0198^T^	MK849797	MK828651	N/A	N/A	Luo et al. (2019)
*D.longispora*	HFJAU 0705^T^	MH555357	MH555359	N/A	N/A	Song et al. (2020)
*D.longnanensis*	HJAUP C1040^T^	OQ942886	OQ942887	OQ944451	N/A	Hu et al. (2023)
*D.martinii*	CGMCC 3.18651^T^	KX033566	KU999975	N/A	N/A	Xia et al. (2017)
*D.meilingensis*	JAUCC 4727^T^	OK562396	OK562390	OK562408	N/A	Zhai et al. (2022)
*D.menghaiensis*	HJAUP C2045^T^	OQ942900	OQ942890	N/A	N/A	Hu et al. (2023)
	HJAUP C2170^T^	OQ942888	OQ942899	OQ944457	OQ944461	Hu et al. (2023)
*D.mengsongensis*	HJAUP C2126^T^	OP78784	OP787876	OP961937	N/A	Liu et al. (2023)
*D.multiseptata*	MFLUCC 16-1044	MF077555	MF077544	MF135652	MF135644	Yang et al. (2018)
	MFLUCC 15-0609^T^	KX710140	KX710145	MF135659	N/A	Hyde et al. (2016)
*D.nabanheensis*	HJAUP C2003^T^	OP787877	OP787873	OP961935	N/A	Liu et al. (2023)
*D.nanchangensis*	HJAUP C1074^T^	OQ942895	OQ942889	OQ944454	OQ944460	Hu et al. (2023)
*D.neorostrata*	MFLUCC 18-0376^T^	MN163017	MN163008	N/A	N/A	Luo et al. (2019)
*D.nonrostrata*	KUNCC 21-10730^T^	OK341198	OK310699	OP413481	OP413475	Zhang et al. (2022)
*D.obclavata*	MFLUCC 18-0329^T^	MN163010	MN163012	N/A	N/A	Luo et al. (2019)
*D.obpyriformis*	MFLUCC 17-1694^T^	MG979764	N/A	MG988422	MG988415	Luo et al. (2018)
	DLUCC 0867	MG979765	MG979757	MG988423	MG988416	Luo et al. (2018)
*D.pachyconidia*	KUMCC 21-10724^T^	OK341194	OK310696	OP413477	OP413471	Zhang et al. (2022)
*D.palmarum*	MFLUCC 18-1446^T^	MK079663	MK085062	MK087660	MK087670	Hyde et al. (2019)
*D.phangngaensis*	MFLUCC 16-0857^T^	MF077556	MF077545	MF135653	N/A	Yang et al. (2018)
*D.phragmiticola*	GUCC 22-0202^T^	OP749881	OP749888	OP749892	OP752700	Hyde et al. (2023)
*D.rayongensis*	MFLUCC 18-0415^T^	MH457137	MH457172	MH463253	MH463255	Hyde et al. (2020)
	MFLUCC 18-0417	MH457138	MH457173	MH463254	MH463256	Hyde et al. (2020)
*D.rostrata*	MFLUCC 16-0969^T^	MG979766	MG979758	MG988424	MG988417	Luo et al. (2018)
	DLUCC 0885	MG979767	MG979759	MG988425	N/A	Luo et al. (2018)
*D.saprophytica*	MFLUCC 18-1238^T^	MW287780	MW286506	MW396651	MW504069	Dong et al. (2021)
*D.septata*	GZCC 22-0078^T^	ON527947	ON527939	ON533683	ON533690	Ma et al. (2022)
*D.sinensis*	HJAUP C2044^T^	OP787875	OP787878	OP961936	N/A	Liu et al. (2023)
*D.songkhlaensis*	MFLUCC 18-1234^T^	MW287755	MW286482	MW396642	N/A	Dong et al. (2021)
*D.suoluoensis*	MFLUCC 17-0224^T^	MF077557	MF077546	MF135654	N/A	Yang et al. (2018)
	MFLUCC 17-1305	MF077558	MF077547	N/A	N/A	Yang et al. (2018)
*D.tectonae*	MFLUCC 12-0291^T^	KX751713	KX751711	KX751710	KX751708	Hyde et al. (2016)
	MFLU 20-0262	MT232719	MT232714	N/A	N/A	Sun et al. (2020)
	MFLUCC 16-0946	MG979768	MG979760	MG988426	MG988418	Dong et al. (2021)
***D.tectonae***	**GZCC 22**-**2046**	**OR348896**	**OR427330**	**OR449124**	**OR449121**	**This study**
*D.tectonigena*	MFLUCC 12-0292^T^	KX751714	KX751712	N/A	KX751709	Hyde et al. (2016)
*D.thailandica*	MFLUCC 16-0270^T^	MH260292	MH275060	MH412767	N/A	Tibpromma et al. (2018)
*D.thysanolaenae*	KUN-HKAS 102247^T^	MK064091	MK045851	MK086031	N/A	Phukhamsak et al. (2019)
*D.tropica*	GZCC 22-0076^T^	ON527943	ON527935	ON533679	ON533687	Ma et al. (2022)
*D.verrucosa*	GZCC20-0434^T^	MZ868762	MZ868771	MZ892977	MZ892983	Yang et al. (2021)
*D.wuzhishanensis*	GZCC 22-0077^T^	ON527946	ON527938	ON533682	N/A	Ma et al. (2022)
*D.xishuangbannaensis*	KUMCC 17-0290^T^	MH260293	MH275061	MH412768	MH412754	Tibpromma et al. (2018)
*D.yichunensis*	HJAUP C1065^T^	OQ942892	OQ942885	OQ944453	OQ944459	Hu et al. (2023)
*D.yongxiuensis*	JAUCC 4725^T^	OK562394	OK562388	OK562406	N/A	Zhai et al. (2022)
*D.yunjushanensis*	JAUCC 4723^T^	OK562398	OK562392	OK562410	N/A	Zhai et al. (2022)
*D.yunnanensis*	MFLUCC 20-0153^T^	MW081546	MW081541	MW084995	MW151861	Li et al. (2021)

Note: “^T^” denotes ex-type strain. Newly generated sequences are indicated in black bold. “N/A”: no data available in GenBank.

The phylogenetic analyses were conducted using the CIPRES Science Gateway V. 3.3. “RAxML-HPC v.8 on XSEDE”, “PAUP on XSEDE”, and “MrBayes on XSEDE (3.2.7a)” were utilized for ML, MP, and BYPP methods, respectively (Huelsenbeck and Ronquist 2001; Swofford 2002; Stamatakis et al. 2008; Miller et al. 2010; Ronquist et al. 2012). For the ML analysis, the GTRGAMMA model of nucleotide evolution was employed, and RAxML rapid bootstrapping with 1,000 bootstrap replicates was obtained (Stamatakis et al. 2008).

The MP analysis employed 1,000 random taxa additions to infer trees. Branches of zero length were collapsed, and all multiple parsimonious trees were saved. The maxtrees value was set to 5,000. For trees generated using different optimal criteria, parsimony score values were determined for tree length (TL), consistency index (CI), retention index (RI), and homoplasy index (HI). To assess clade stability, the bootstrap (BT) method was used with 1,000 iterations, each consisting of 100 trials of random stepwise addition of taxa (Hillis and Bull 1993).

The posterior probabilities (PP) were determined based on Bayesian Markov chain Monte Carlo sampling (Huelsenbeck and Ronquist 2001). The best nucleotide substitution model for each data partition was determined using the program MrModeltest 2.2 (Nylander 2004). The GTR + I + G substitution model with gamma rates and Dirichlet base frequencies was selected for all LSU, ITS, *tef*1-α, and *rpb*2 sequences. To calculate the posterior probabilities, four simultaneous Markov chains were run for one million generations, with trees sampled every 100^th^ generation, resulting in a total of 10,000 trees. A burn-in parameter of 0.25 was set, indicating that 75% of the trees were remined during the burn-in phase, and the remaining trees were used for calculating the posterior probabilities in the majority rule consensus tree.

FigTree v. 1.4.4. was used for visualizing the phylogenetic trees, and Adobe Illustrator CC 2019v. 23.1.0 was used to edit trees and figure layout.

### ﻿Phylogenetic analyses results

**Figure 1.**
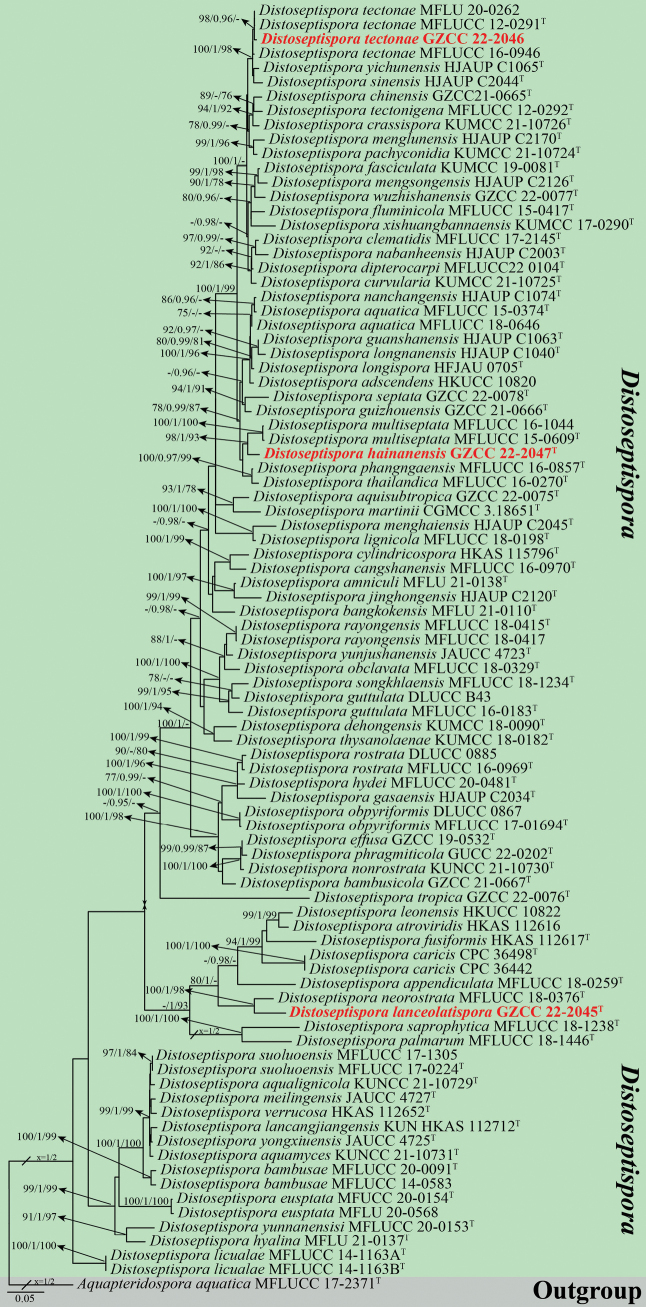
Phylogenetic tree generated from ML analysis based on a combination of LSU, ITS, *tef*1-a, and *rpb*2 sequence data. Bootstrap support values of ML and MP equal to or greater than 75%, and PP value equal to or greater than 0.95 are given near the nodes as ML/PP/MP. The tree is rooted with *Aquapteridosporaaquatica* (MFLUCC 17-2371). Ex-type strains are indicated by the superscript T. The new collections are in bold red text.

This study utilized a combined multi-gene dataset encompassing ITS, LSU, *tef*1-α, and *rpb*2 sequences to assess the phylogenetic relationships among *Distoseptispora* species. The analyses included a total of 90 taxa, designating *Aquapteridosporaaquatica* X.D. Yu, W. Dong & H. Zhang (MFLUCC 17-2371) as the outgroup taxon. The combined aligned sequence matrix comprised 3,360 characters, including gaps: LSU (1–840 bp), ITS (841–1406 bp), *tef*1-α (1407–2321 bp), and *rpb*2 (2322–3360 bp). The ML, MP, and Bayesian trees analyzed exhibited a high degree of similarity in topology and showed no significant conflicts. The RAxML analysis yielded a best-scoring tree (ln = -31666.963504), which is presented in Fig. 1. The matrix encompassed 1572 distinct alignment patterns, with 27.15% constituted by undetermined characters or gaps. The estimated base frequencies were as follows: A = 0.239306, C = 0.265297, G = 0.281926, T = 0.213472; substitution rates AC = 1.429077, AG = 3.512798, AT = 1.204511, CG = 0.845859, CT = 6.948345, GT = 1.000000; gamma distribution shape parameter *α* = 0.244431. For the MP analysis, 3360 characters remained unchanged, 330 were variable and parsimoniously uninformative, and 1074 were parsimoniously informative. The most parsimonious tree yielded the following values: TL = 5624, CI = 0.400, RI = 0.738, RC = 0.295, HI = 0.600. For BYPP analysis, Bayesian posterior probabilities from MCMC were evaluated with a final average standard deviation of split frequencies of 0.009754.

In the phylogenetic analyses (Fig. 1), all our newly identified taxa nested within *Distoseptispora*, affirming their classification within this genus. *Distoseptisporahainanensis* (GZCC 22-047) formed a sister clade to *D.multiseptata* strains (MFLUCC 16-1044 and MFLUCC 15-0609) with 98% ML, 1.00 PP, and 93% MP statistical support. *Distoseptisporalanceolatispora* (GZCC 22-2045) formed a sister clade to *D.neorostrata* (MFLUCC 18-0376) with 100% ML, 1.00 PP, and 98% MP statistical support. In addition, our new collection GZCC 22-2046 clustered together with three *D.tectonae* strains (MFLU 20-0262 and MFLUCC 12-0291) with 98% ML and 0.96 PP statistical support, indicating they represent the same species.

## ﻿Taxonomy

### ﻿ Distoseptispora hainanensis

Taxon classificationFungiDistoseptisporalesDistoseptisporaceae

X.M. Chen & Y.Z. Lu sp. nov.

1369CFAC-E4DC-5C03-8878-CEA7116DE0F3

Index Fungorum: IF900953

Facesoffungi Number: FoF14663

Fig. 2

#### Etymology.

The epithet refers to the location “Hainan Province” where the holotype was collected.

#### Holotype.

GZAAS 22-2047.

#### Description.

***Saprobic*** on decaying wood in terrestrial habitat. **Sexual morph**: Undetermined. **Asexual morph: *Colonies*** on natural substrate superficial, effuse, dark brown, and hairy. ***Mycelium*** mostly immersed, composed of branched, septate, brown to dark brown, smooth hyphae. ***Conidiophores*** 70–130 × 5–8.5 μm (x– = 103 × 7 μm, n = 20), macronematous, mononematous, erect, solitary, straight or slightly flexuous, brown to dark brown, paler towards the apex, cylindrical, 4–6-septate, slightly constricted and darkened at septa, unbranched, thick-walled. ***Conidiogenous cells*** 6–13 × 3.5–6.5 μm (x– = 10 × 5 μm, n = 20), holoblastic, monoblastic, integrated, terminal, indeterminate, cylindrical, slightly tapering towards the apex, brown, percurrent. ***Conidia*** 44–117 μm × 9–18.5 μm (x– = 90 × 14 μm, n = 20), acrogenous, solitary, obclavate or obpyriform, rostrate, truncate at the base, straight or slightly curved, up to 22-distoseptate, slightly constricted at septa, brown, verrucose.

**Figure 2.**
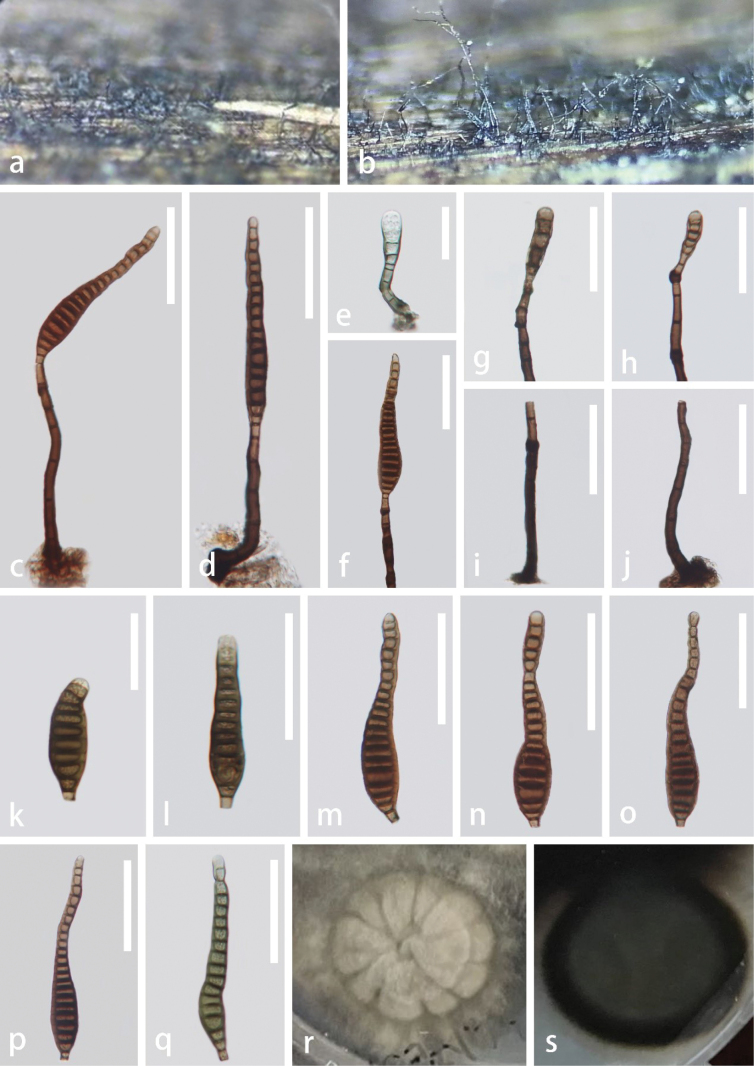
*Distoseptisporahainanensis* (GZAAS 22-2047, holotype) **a, b** colonies on substrate **c–e** conidiophores and conidia **f–h** conidiogenous cells bearing conidia **i, j** conidiophores **k–q** conidia **r, s** colony on PDA (**r** from front **s** from reverse). Scale bars: 50 μm (**c**, **d**, **f–j**, **l–q**); 30 μm (**e, k**).

#### Culture characteristics.

Colonies grown on PDA circular, dense, fluffy, with raised center and lobate edge, pale gray in the center, grayish brown in the outer ring from the front view, dark brown in the center, and blackish brown in the outer ring from the reverse view.

#### Material examined.

China, Hainan Province, on unidentified decaying wood, 15 May 2021, Xia Tang, HN02 (GZAAS 22-2047, holotype), ex-type living culture, GZCC 22-2047.

#### Notes.

Morphologically, *Distoseptisporahainanensis* is similar to *D.effusa* L.L. Liu & Z.Y. Liu in having macronematous conidiophores, monoblastic conidiogenous cells, and acrogenous, obclavate, rostrate conidia (Yang et al. 2021). However, conidia of *D.hainanensis* are up to 22-distoseptate, whereas those of *D.effusa* are only 4–9-distoseptate. In the phylogenetic analyses, *D.hainanensis* formed a distinct clade sister to *D.multiseptata* Jiao Yang & K.D. Hyde with 98% ML, 1 PP, and 93% MP statistical support (Fig. 1). *Distoseptisporahainanensis* differs from *D.multiseptata* in having brown, longer conidiophores (70–130 μm *vs.* 23–65 µm) and obclavate or obpyriform, brown, verrucose, smaller conidia (44–117 μm *vs.* up to 290 µm) (Hyde et al. 2016). Comparing DNA sequence data, *D.hainanensis* diverges from *D.multiseptata* (MFLUCC 15-0609) in the ITS by 21/552 bp (3.8% difference), in the LSU by 1/812 bp (0.01% difference), in *tef*1-α by 33/912 bp (3.6% difference), and no data is available for *rpb*2 of *D.multiseptata* (MFLUCC 15-0609) in GenBank. Hence, the novel species, *D.hainanensis*, is introduced, following the guidelines of Jeewon and Hyde (2016) and Chethana et al. (2021).

### ﻿ Distoseptispora lanceolatispora

Taxon classificationFungiDistoseptisporalesDistoseptisporaceae

X.M. Chen & Y.Z. Lu sp. nov.

558A79C2-EC0D-5BBD-BFB7-91B2EE3E304A

Index Fungorum: IF900954

Facesoffungi Number: FoF14664

Fig. 3

#### Etymology.

Referring to the lanceolate conidia.

#### Holotype.

GZAAS 22-2045.

#### Description.

***Saprobic*** on submerged decaying wood in freshwater habitat. **Sexual morph**: Undetermined. **Asexual morph: *Colonies*** on substrate effuse, gregarious, hairy, pale brown to brown. ***Mycelium*** mostly immersed, composed of septate, yellow-brown to brown, smooth hyphae. ***Conidiophores*** 120–190 × 4–8 µm (x– = 155 × 6.5 µm, n = 20), macronematous, mononematous, erect, solitary, straight or slightly flexuous, grayish brown to dark brown, slightly tapering towards the apex, cylindrical, 7–8-septate, unbranched, thick-walled, smooth-walled. ***Conidiogenous cells*** 15–27 × 3–5.5 µm (x– = 20.5 × 4.5 µm, n = 20), monoblastic, integrated, terminal, cylindrical, slightly tapering towards the apex, pale brown, percurrent. ***Conidia*** 31–90 × 9.5–15 µm (x– = 58.5 × 13 µm, n = 20), acrogenous, solitary, fusiform or lanceolate, rostrate, truncate at the base, straight or slightly curved, 5–13-distoseptate, slightly constricted at septa, olivaceous to olivaceous brown, slightly paler at the apex, verrucous, with or without apical, hyalina appendages.

**Figure 3.**
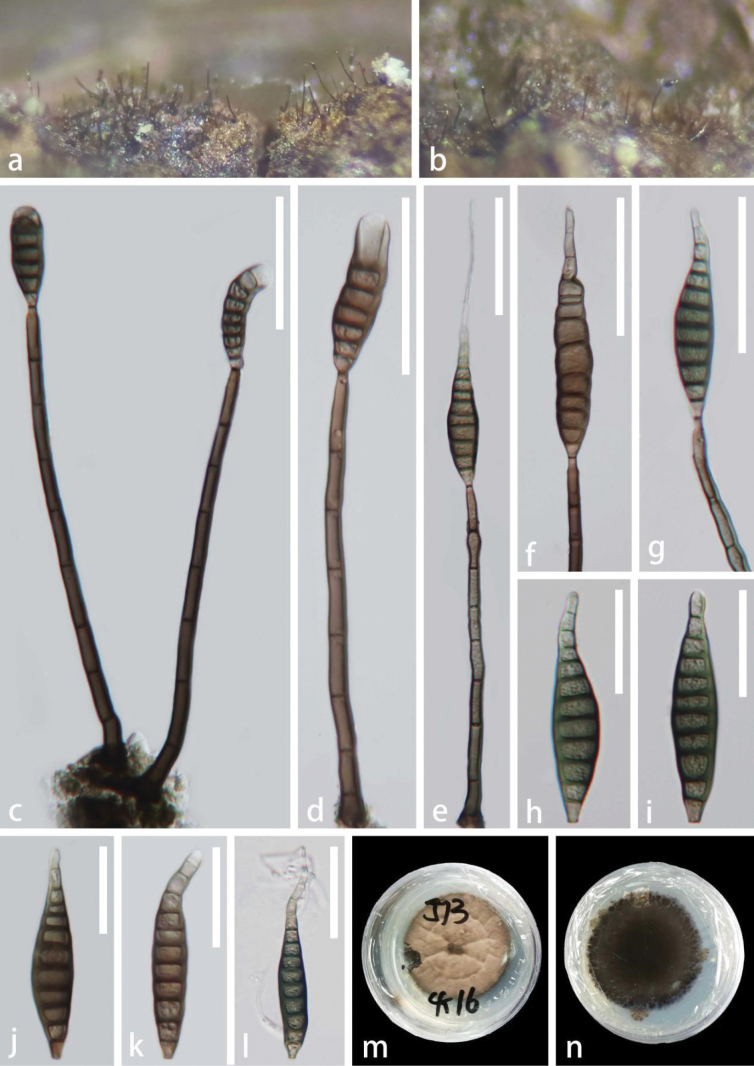
*Distoseptisporalanceolatispora* (GZAAS 22-2045, holotype) **a, b** colonies on substrate **c–e** conidiophores and conidia **f, g** conidiogenous cells bearing conidia **h–k** conidia **l** germinated conidium **m, n** colony on PDA (**m** from front **n** from reverse). Scale bars: 50 μm (**c–g**); 30 μm (**h–l**).

#### Culture characteristics.

Colonies grown on PDA circular, dense, flat, dry, gray to dark gray, radially striated, and a ring in the middle of the colonies with an entire edge from the front view, dark brown to black with a circular, gray edge from reverse view, not pigmented.

#### Material examined.

China, Hainan Province, on submerged decaying wood in a freshwater stream, 23 October 2021, Jian Ma, J13 (GZAAS 22-2045, holotype), ex-type living culture, GZCC 22-2045.

#### Notes.

*Distoseptisporalanceolatispora* is morphologically similar to *D.leonensis* (M.B. Ellis) R. Zhu & H. Zhang. However, compared to *D.lanceolatispora*, *D.leonensis* has longer conidiophores (120–190 µm vs. 110–130 µm), longer conidiogenous cells (15–27 µm *vs.* 5–15 µm), and 5–13-distoseptate, fusiform or lanceolate conidia (Zhang et al. 2022). In the phylogenetic analyses (Fig. 1), *D.lanceolatispora* forms a unique clade adjacent to *D.neorostrata* D.F. Bao, Z.L. Luo & H.Y. Su with 100% ML, 1 PP, and 98% MP support. Based on a pairwise nucleotide comparison of ITS and LSU sequences, *D.lanceolatispora* deviates from *D.neorostrata* by 39/529 bp (6.8%) for ITS and 14/850 bp (1.6%) for LSU, and there is no data available for *tef*1-α and *rpb*2 for *D.neorostrata* (MFLUCC 18-0376) in GenBank. Hence, we introduce the new species, *D.lanceolatispora*, based on the criteria established by Jeewon and Hyde (2016) and Chethana et al. (2021).

### ﻿ Distoseptispora tectonae

Taxon classificationFungiDistoseptisporalesDistoseptisporaceae

Doilom & K.D. Hyde, Fungal Diversity 80: 222 (2016)

E502DE4A-6E52-505B-B0FD-A7463654155E

Index Fungorum: IF552223

Facesoffungi number: FoF01877

Fig. 4

#### Description.

***Saprobic*** on dead twigs of *Edgeworthiachrysantha*. **Sexual morph**: Undetermined. **Asexual morph: *Colonies*** on natural substrate abundant, superficial, dark brown, hairy. ***Conidiophores*** 35–80 μm × 4–7.5 μm (x– = 58 × 5.5 μm, n = 20), macronematous, mononematous, simple, erect to slightly curved, solitary, pale brown to dark brown, cylindrical, 2–4-septate, slightly constricted at the septa, unbranched, thick-walled. ***Conidiogenous cells*** 6–10 μm × 3.5–6.5 μm (x– = 8 × 4.5 μm, n = 20), holoblastic, monoblastic, integrated, terminal, cylindrical, slightly tapering towards the apex, brown to reddish brown, percurrent. ***Conidia*** 190–255 μm × 9.5–16 μm (x– = 220 μm × 13 μm, n = 20), 5–16 μm (x– = 13 μm, n = 20) wide at the protruding truncate base; 4.5–8 μm (x– = 6.5 μm, n = 20) wide in the tapering part, acrogenous, solitary, obclavate, elongate, rostrate, straight or curved, tapering towards the apex, 9–39-distoseptate, olivaceous-green when young, dark reddish brown at maturity, verrucose.

**Figure 4.**
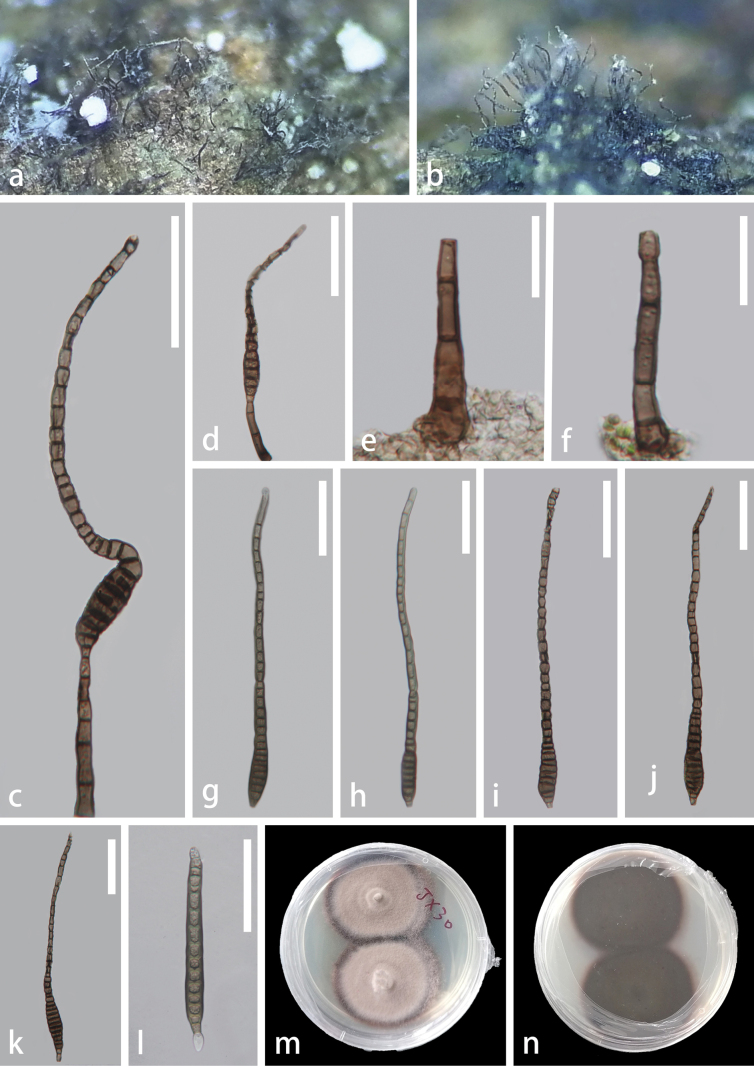
*Distoseptisporatectonae* (GZAAS 22-2046) **a**, **b** colonies on substrate **c, d** conidiophores and conidia **e, f** conidiophores **g–k** conidia **l** germinated conidium **m, n** colonies on PDA (**m** from front **n** from reverse) Scale bars: 50 μm (**c, d, g–l**); 20 μm (**e, f**).

#### Culture characteristics.

Conidia germinating on PDA within 24 h, colonies circular, dense, umbonate, spreading, fluffy. The surface is slightly rough with reddish-gray mycelium, colonies somewhat raised in the middle, and with a filiform edge. The reverse side is dark gray with a circular, pale reddish-gray edge, not pigmented.

#### Material examined.

China, Guizhou Province, Guiyang City, Guiyang Medicinal Botanical Garden, on dead twigs of *Edgeworthiachrysantha*, 20 August 2022, Xia Tang, JX30 (GZAAS 22-2046), living culture, GZCC 22-2046.

#### Known host and distribution.

*Tectonagrandis* (Thailand, Hyde et al. 2016), on dead stems (Thailand, Sun et al. 2020), on dead, submerged, decaying wood of unidentified plants (China & Thailand, Luo et al. 2019; Dong et al. 2021; Zhang et al. 2022), and dead twig and branch of *Edgeworthiachrysantha* (China, this study).

#### Notes.

*Distoseptisporatectonae* was first isolated from a dead twig of *Tectonagrandis* in Thailand (Hyde et al. 2016). Since then, this species has been identified in various countries on different substrates and hosts (Hyde et al. 2016; Sun et al. 2020; Dong et al. 2021; Zhang et al. 2022). In the phylogenetic tree (Fig. 1), our new isolate forms a close lineage to *D.tectonae* (GZCC 22-2046) with statistical support of 98% ML and 0.96 PP. Based on pairwise nucleotide comparisons of ITS, LSU, *tef*1-α, and *rpb*2, our new isolate diverges from *D.tectonae* (MFLUCC 12-0291, ex-type) by 6/554 bp (1%) for ITS, 1/852 bp (0.01%) for LSU, 0/980 bp (0%) for *tef*1-α, and 2/899 bp (0.2%) for *rpb*2. In addition, the morphological characteristics of our isolate match well with the holotype description of *D.tectonae* (Hyde et al. 2016). This study reports a new host record of *Distoseptisporatectonae* on dead twigs of *Edgeworthiachrysantha* in China.

## ﻿Discussion

*Distoseptispora* is one of the sporidesmium-like taxa and is well-known for its asexual morph, which has considerable morphological variations (Su et al. 2016; Yang et al. 2018, 2021). However, the phylogenetic analyses suggest a lack of correlation between phylogenetic relationships and morphological analyses. For instance, species such as *D.appendiculata* D.F. Bao, Z.L. Luo & H.Y. Su, *D.atroviridis* J. Yang & K.D. Hyde, *D.caricis* Crous, *D.fusiformis* J. Yang & K.D. Hyde, *D.lanceolatispora*, *D.leonensis*, *D.neorostrata*, *D.palmarum* S.N. Zhang, K.D. Hyde & J.K. Liu, and *D.saprophytica* W. Dong, H. Zhang & K.D. Hyde cluster together as a subclade in the phylogenetic tree (see Fig. 1). In contrast, morphological analysis reveals significant differences, especially in the characteristics of conidiophores, conidiogenous cells, and conidia (Crous et al. 2019; Hyde et al. 2019; Luo et al. 2019; Dong et al. 2021; Yang et al. 2021; Zhang et al. 2022). This disparity is common within the genus. We recommend adopting a combination approach using molecular and morphological methods for more effective identification within this genus.

Worth noting, among the various species of *Distoseptispora*, *D.martinii* (J.L. Crane & Dumont) J.W. Xia & X.G. Zhang stands out due to its unique morphological characteristics, especially its oblate or subglobose conidia, distinguishing it from other species within *Distoseptispora* (Xia et al. 2017). The species was initially introduced as *Acrodictysmartinii* J.L. Crane & Dumont by Crane and Dumont (1975) based on morphological characteristics. Then, it underwent several taxonomic revisions based solely on morphology (Baker et al. 2002; Delgado 2009). Later, Xia et al. (2017) reclassified *Acrodictysmartinii* as *D.martinii* based on genetic analysis. However, the morphological traits of *D.martinii* greatly diverge from typical *Distoseptispora* features (Crane and Dumont 1975; Xia et al. 2017). Therefore, we suggest additional collections and analysis of *D.martinii* specimens to ensure the reliability of the provided DNA sequence data.

In recent years, *Distoseptispora* species have been reported worldwide, such as in China, Hungary, Hawaii, Malaysia, and Thailand (Shoemaker and White 1985; McKenzie 1995; Wu and Zhuang 2005; Zhang et al. 2022). Studies on *Distoseptispora* have been particularly extensive in China and Thailand (Hyde et al. 2016, 2019, 2020; Su et al. 2016; Yang et al. 2018, 2021; Luo et al. 2019; Sun et al. 2020; Hu et al. 2023). To date, 73 species of *Distoseptispora* have been documented, of which 55 have been recorded in China (including known species, see Table 2). Our collections further highlight the distribution of the genus in China, and we speculate that the country may harbor a greater diversity of the genus. Thus, future studies are needed to validate this hypothesis.

**Table 2.** *Distoseptispora* species and their locations, lifestyles, habitats, hosts, and corresponding references.

Species	Country	Habitat	Host	References
*D.adscendens*	China; Hungary; Hawaii	Terrestrial	Decaying wood and decaying branches of many woody plant species; *Platanusorientalis*	Shoemaker et al. (1985); McKenzie et al. (1995); Wu et al. (2005); Zhang et al. (2022)
*D.amniculi*	Thailand	Freshwater	Submerged decaying wood	Yang et al. (2021)
*D.appendiculata*	Thailand	Freshwater	Submerged decaying wood	Luo et al. (2019)
*D.aqualignicola*	China	Freshwater	Submerged decaying wood	Zhang et al. (2022)
*D.aquamyces*	China	Freshwater	Submerged decaying wood	Zhang et al. (2022)
*D.aquatica*	China	Freshwater	Submerged decaying wood	Su et al. (2016); Luo et al. (2019); Li et al. (2021)
*D.aquisubtropica*	China	Freshwater	Submerged decaying wood	Ma et al. (2022)
*D.atroviridis*	China	Freshwater	Submerged decaying wood	Yang et al. (2021)
*D.bambusae*	China	Terrestrial	Decaying bamboo culms	Sun et al. (2020)
*D.bambusicola*	China	Freshwater	Submerged bamboo culms	Jayawardena et al. (2022)
*D.bangkokensis*	Thailand	Freshwater	Submerged decaying wood	Shen et al. (2021)
*D.cangshanensis*	China	Freshwater	Submerged decaying wood	Luo et al. (2018)
*D.caricis*	Thailand	Terrestrial	Leaves of *Carex* sp.	Crous et al. (2019)
*D.chinensis*	China	Freshwater	Submerged decaying wood	Hyde et al. (2021)
*D.clematidis*	China; Thailand	Freshwater; Terrestrial	Dried stem of *Clematissikkimensis*; submerged decaying wood	Phukhamsakda et al. (2020); Shen et al. (2021)
*D.crassispora*	China	Freshwater	Submerged decaying wood	Zhang et al. (2022)
*D.curvularia*	China	Freshwater	Submerged decaying wood	Zhang et al. (2022)
*D.cylindricospora*	China	Freshwater	Submerged decaying wood	Phukhamsakda et al. (2022)
*D.dehongensis*	China; Thailand	Freshwater	Submerged decaying wood	Hyde et al. (2019); Zhang et al. (2022)
*D.dipterocarpi*	Thailand	Terrestrial	Woody litter of *Dipterocarpus* sp.	Afshari et al. (2023)
*D.effusa*	China	Freshwater	Submerged decaying wood	Yang et al. (2021)
*D.euseptata*	China	Freshwater	Submerged decaying wood	Li et al. (2021)
*D.fasciculata*	Thailand	Freshwater	Submerged decaying wood	Dong et al. (2021)
*D.fluminicola*	China	Freshwater	Submerged decaying wood	Su et al. (2016); Luo et al. (2018)
*D.fusiformis*	China	Freshwater	Submerged decaying wood	Yang et al. (2021)
*D.gasaensis*	China	Terrestrial	Decaying branches of broadleaf tree	Hu et al. (2023)
*D.guanshanensis*	China	Terrestrial	Decaying branches of broadleaf tree	Hu et al. (2023)
*D.guizhouensis*	China	Terrestrial	Decaying wood	Hyde et al. (2021)
*D.guttulata*	Thailand	Freshwater	Submerged decaying wood	Yang et al. (2018); Luo et al. (2019)
*D.hainanensis*	China	Terrestrial	Decaying wood	This study
*D.hyalina*	Thailand	Freshwater	Submerged decaying wood	Yang et al. (2021)
*D.hydei*	Thailand	Terrestrial	Decaying bamboo culms	Monkai et al. (2020)
*D.jinghongensis*	China	Terrestrial	Decaying branches of broadleaf tree	Hu et al. (2023)
*D.lancangjiangensis*	China	Freshwater	Submerged decaying wood	Shen et al. (2021)
*D.lanceolatispora*	China	Freshwater	Submerged decaying wood	This study
*D.leonensis*	China; Malaysia	Terrestrial	Decaying culms of grasses or decaying branches	McKenzie et al. (1995); Wu et al. (2005); Zhang et al. (2022)
*D.licualae*	Thailand	Terrestrial	Decaying leaves of *Licualaglabra*	Konta et al. (2023)
*D.lignicola*	China; Thailand	Freshwater	Submerged decaying wood	Luo et al. (2019); Yang et al. (2021)
*D.longispora*	China	Freshwater	Submerged decaying wood	Song et al. (2020)
*D.longnanensis*	China	Terrestrial	Decaying branches of broadleaf tree	Hu et al. (2023)
*D.martinii*	China	Terrestrial	Decaying branches	Xia et al. (2017)
*D.meilingensis*	China	Freshwater	Decaying bamboo culms	Zhai et al. (2022)
*D.menghaiensis*	China	Terrestrial	Decaying branches of broadleaf tree	Hu et al. (2023)
*D.menglunensis*	China	Terrestrial	Decaying branches of broadleaf tree	Hu et al. (2023)
*D.mengsongensis*	China	Terrestrial	Decaying branches	Liu et al. (2023)
*D.multiseptata*	Thailand	Freshwater	Submerged decaying wood	Hyde et al. (2016); Yang et al. (2018)
*D.nabanheensis*	China	Terrestrial	Decaying branches	Liu et al. (2023)
*D.nanchangensis*	China	Terrestrial	Decaying branches of broadleaf tree	Hu et al. (2023)
*D.neorostrata*	Thailand	Freshwater	Submerged decaying wood	Luo et al. (2019)
*D.nonrostrata*	China	Freshwater	Submerged decaying wood	Zhang et al. (2022)
*D.obclavata*	Thailand	Freshwater	Submerged decaying wood	Luo et al. (2019)
*D.obpyriformis*	China	Freshwater	Submerged decaying wood	Luo et al. (2018)
*D.pachyconidia*	China	Freshwater; Terrestrial	Submerged decaying wood; decaying wood	Ma et al. (2022); Zhang et al. (2022)
*D.palmarum*	Thailand	Terrestrial	Rachis of *Cocosnucifera*	Hyde et al. (2019)
*D.phangngaensis*	Thailand	Freshwater	Submerged decaying wood	Yang et al. (2018)
*D.phragmiticola*	China	Terrestrial	Decaying *Phragmitesaustralis*	Hyde et al. (2023)
*D.rayongensis*	Thailand	Freshwater	Submerged decaying wood	Hyde et al. (2020)
*D.rostrata*	China	Freshwater	Submerged decaying wood	Luo et al. (2018)
*D.saprophytica*	Thailand	Freshwater	Submerged decaying wood	Dong et al. (2021)
*D.septata*	China	Freshwater	Submerged decaying wood	Ma et al. (2022)
*D.sinensis*	China	Terrestrial	Decaying branches	Liu et al. (2023)
*D.songkhlaensis*	Thailand	Freshwater	Submerged decaying wood	Dong et al. (2021)
*D.suoluoensis*	China	Freshwater	Submerged decaying wood	Yang et al. (2018)
*D.tectonae*	China; Thailand	Terrestrial; Freshwater	Decaying twig of *Tectonagrandis*; stems of dead wood; submerged decaying wood; decaying twigs of *Edgeworthiachrysantha*	Hyde et al. (2016); Luo et al. (2018); Sun et al. (2020); Dong et al. (2021); Li et al. (2021); Zhang et al. (2022); This study
*D.tectonigena*	Thailand	Terrestrial	Decaying twig of *Tectonagrandis*	Hyde et al. (2016)
*D.thailandica*	Thailand	Terrestrial	Decaying leaves of *Pandanus* sp.	Tibpromma et al. (2018)
*D.thysanolaenae*	China	Terrestrial; Freshwater	Decaying culms of *Thysanolaenamaxima*; Submerged decaying wood	Phookamsak et al. (2019); Shen et al. (2021)
*D.tropica*	China	Terrestrial	Decaying wood	Ma et al. (2022)
*D.verrucosa*	China	Freshwater	Submerged decaying wood	Yang et al. (2021)
*D.wuzhishanensis*	China	Freshwater	Submerged decaying wood	Ma et al. (2022)
*D.xishuangbannaensis*	China	Terrestrial; Freshwater	Decaying leaves of *Pandanusutilis*; submerged decaying wood	Tibpromma et al. (2018); Ma et al. (2022)
*D.yichunensis*	China	Terrestrial	Decaying branches of broadleaf tree	Hu et al. (2023)
*D.yongxiuensis*	China	Freshwater	Decaying bamboo culms	Zhai et al. (2022)
*D.yunjushanensis*	China	Freshwater	Decaying bamboo culms	Zhai et al. (2022)
*D.yunnanensis*	China	Freshwater	Submerged decaying wood	Li et al. (2021)

## Supplementary Material

XML Treatment for Distoseptispora hainanensis

XML Treatment for Distoseptispora lanceolatispora

XML Treatment for Distoseptispora tectonae

## ﻿Additional information

### Author ORCIDs

Xue-Mei Chen https://orcid.org/0009-0004-8631-0735

Xia Tang https://orcid.org/0000-0003-2705-604X

Jian Ma https://orcid.org/0009-0008-1291-640X

Ning-Guo Liu https://orcid.org/0000-0002-9169-2350

Saowaluck Tibpromma https://orcid.org/0000-0002-4706-6547

Samantha C. Karunarathna https://orcid.org/0000-0001-7080-0781

Yuan-Pin Xiao https://orcid.org/0000-0003-1730-3545

Yong-Zhong Lu https://orcid.org/0000-0002-1033-5782

### Data availability

All of the data that support the findings of this study are available in the main text.

## References

[B1] Afshari N Gomes de Farias AR Bhunjun CS Phukhamsakda C Hyde KD Lumyong S (2023) *Distoseptisporadipterocarpi* sp. nov. (Distoseptisporaceae), a lignicolous fungus on decaying wood of *Dipterocarpus* in Thailand. Current Research in Environmental & Applied Mycology 13(1): 68–78. 10.5943/cream/13/1/5

[B2] Baker WA Partridge EC Morgan-Jones G (2002) Notes on hyphomycetes LXXXV. *Junewangia*, a genus in which to classify four *Acrodictys* species and a new taxon. Mycotaxon 81: 293–319.

[B3] Chethana KWT Manawasinghe IS Hurdeal VG Bhunjun CS Appadoo MA Gentekaki E Raspé O Promputtha I Hyde KD (2021) What are fungal species and how to delineate them? Fungal Diversity 109(1): 1–25. 10.1007/s13225-021-00483-9

[B4] Crane JL Dumont KP (1975) Hyphomycetes from the West Indies and Venezuela. Canadian Journal of Botany 53(9): 843–851. 10.1139/b75-102

[B5] Crous PW Wingfield MJ Lombard L Roets F Swart WJ Alvarado P Carnegie AJ Moreno G Luangsaard J Thangavel R Alexandrova AV Baseia IG Bellanger JM Bessette AJ Bessette AR Peña-Lastra SDL García D Gené J Pham THG Heykoop M Malysheva E Malysheva V Martín MP Morozova OV Noisripoom W Overton BE Rea AE Sewall BJ Smith ME Smyth CW Tasanathai K Visagie CM Adamčík S Alves A Andrade JP Aninat MJ Araújo RVB Bordallo JJ Boufleur T Baroncelli R Barreto RW Bolin J Cabero J Caboň M Cafà G Caffot MLH Cai L Carlavilla JR Chávez R Castro RRLD Delgat L Deschuyteneer D Dios MM Domínguez LS Evans HC Eyssartier G Ferreira BW Figueiredo CN Liu F Fournier J Galli-Terasawa LV Gil-Durán C Glienke C Gonçalves MFM Gryta H Guarro J Himaman W Hywel-Jones N Iturrieta-González I Ivanushkina NE Jargeat P Khalid AN Khan J Kiran M Kiss L Kochkina JA Kolařík M Kubátová A Lodge DJ Loizides M Luque D Manjón JL Marbach PAS Massola NS Mata M Miller AN Mongkolsamrit S Moreau PA Morte A Mujic A Navarro-Ródenas A Németh MZ Nóbrega TF Nováková A Olariaga I Ozerskaya SM Palma MA Petters-Vandresen DAL Piontelli E Popov ES Rodríguez A Requejo Ó Rodrigues ACM Rong IH Roux J Seifert KA Silva BDB Sklenář F Smith JA Sousa JO Souza HG Souza JTD Švec K Tanchaud P Tanney JB Terasawa F Thanakitpipattana D Torres-Garcia D Vaca I Vaghefi N Iperen ALV Vasilenko OV Verkbeen A Yilmaz N Zamora JC Zapata M Jurjević Ž Groenewald JZ (2019) Fungal Planet description sheets: 951–1041. Persoonia 43(1): 223–425. 10.3767/persoonia.2019.43.06 32214501 PMC7085856

[B6] Cubeta MA Echandi E Abernethy T Vilgalys R (1991) Characterization of anastomosis groups of binucleate *Rhizoctonia* species using restriction analysis of an amplified ribosomal RNA gene. Phytopathology 81(11): 1395–1400. 10.1094/Phyto-81-1395

[B7] Delgado G (2009) South Florida microfungi: *Veramycellabispora*, a new palmicolous, anamorphic genus and species, with some new records for the continental USA. Mycotaxon 107(1): 357–373. 10.5248/107.357

[B8] Dong W Hyde KD Jeewon R Doilom M Yu XD Wang GN Liu NG Hu DM Nalumpang S Zhang H (2021) Towards a natural classification of annulatascaceae-like taxa II: Introducing five new genera and eighteen new species from freshwater. Mycosphere 12(1): 1–88. 10.5943/mycosphere/12/1/1

[B9] Hall TA (1999) BioEdit: A user-friendly biological sequence alignment editor and analysis program for windows 95/98/NT. Nucleic Acids Symposium Series 41: 95–98. 10.1021/bk-1999-0734.ch008

[B10] Hillis DM Bull JJ (1993) An empirical test of bootstrapping as a method for assessing confidence in phylogenetic analysis. Systematic Biology 42(2): 182–192. 10.1093/sysbio/42.2.182

[B11] Hu YF Liu JW Luo XX Xu ZH Xia JW Zhang XG Castañeda-Ruíz RF Ma J (2023) Multi-locus phylogenetic analyses reveal eight novel species of *Distoseptispora* from southern China. Microbiology Spectrum 11(6): e02468–e23. 10.1128/spectrum.02468-23 PMC10715003 37905843

[B12] Huelsenbeck JP Ronquist FJB (2001) MRBAYES: Bayesian inference of phylogenetic trees. Bioinformatics 17(8): 754–755. 10.1093/bioinformatics/17.8.754 11524383

[B13] Hyde KD Hongsanan S Jeewon R Bhat DJ McKenzie EHC Jones EBG Phookamsak R Ariyawansa HA Boonmee S Zhao Q Abdel-Aziz FA Abdel-Wahab MA Banmai S Chomnunti P Cui BK Daranagama DA Das K Dayarathne MC Silva NID Goes-Neto AA Huang SK Jayasiri SC Jayawardena RS Konta S Lee HB Li WJ Lin CG Liu JK Lu YZ Luo ZL Manawasinghe IS Manimohan P Mapook A Niskanen T Norphanphoun C Papizadeh M Perera RH Phukhamsakda C Richter C Santiago ALCMDA Drechsler-Santos ER Senanayake IC Tanaka K Tennakoon TMDS Thambugala KM Tian Q Tibpromma S Thongbai B Vizzini A Wanasinghe DN Wijayawardene NN Wu HX Yang J Zeng XY Zhang H Zhang JF Bulgakov TS Camporesi E Bahkali AH Amoozegar MA Araujo-Neta LS Ammirati JF Baghela A Bhatt RP Bojantchev D Buyck B Silva GAD Lima CLFD Oliveira RJVD Souza CAFD Dai YC Dima B Duong TT Ercole E Mafalda-Freire F Ghosh A Hashimoto A Kamolhan S Kang JC Karunarathna SC Kirk PM Kytovuori I Lantieri A Liimatainen K Liu ZY Liu XY Lucking R Medardi G Mortimer PE Nguyen TTT Promputtha I Raj KNA Reck MA Lumyong S Shahzadeh-Fazeli SA Stadler M Soudi MR Su HY Takahashi T Tangthirasunun N Uniyal P Wang Y Wen TC Xu JC Zhang ZK Zhao YC Zhou JL Zhu L (2016) Fungal diversity notes 367–490: taxonomic and phylogenetic contributions to fungal taxa. Fungal Diversity 80: 1–270. 10.1007/s13225-016-0373-x

[B14] Hyde KD Tennakoon DS Jeewon R Bhat DJ Maharachchikumbura SSN Rossi W Leonardi M Lee HB Mun HY Houbraken J Nguyen TTT Jeon SJ Frisvad JC Wanasinghe DN Lücking R Aptroot A Cáceres MES Karunarathna SC Hongsanan S Phookamsak R Silva NI Thambugala KM Jayawardena RS Senanayake IC Boonmee S Chen J Luo ZL Phukhamsakda C Pereira OL Abreu VP Rosado AWC Bart B Randrianjohany E Hofstetter V Gibertoni TB Soares AWS Plautz Jr HL Sotão HMP Xavier WKS Bezerra JDP Oliveira TGL Souza-Motta CM Magalhães OMC Bundhun D Harishchandra D Manawasinghe IS Dong W Zhang SN Bao DF Samarakoon MC Pem D Karunarathna A Lin CG Yang J Perera RH Kumar V Huang SK Dayarathne MC Ekanayaka AH Jayasiri SC Xiao YP Konta S Niskanen T Liimatainen K Dai YC Ji XH Tian XM Mešić A Singh SK Phutthacharoen K Cai L Sorvongxay T Thiyagaraja V Norphanphoun C Chaiwan N Lu YZ Jiang HB Zhang JF Abeywickrama PD Aluthmuhandiram JVS Brahmanage RS Zeng M Chethana T Wei DP Réblová M Fournier J Nekvindová J Barbosa RN Santos JEF Oliveira NT Li GJ Ertz D Shang QJ Phillips AJL Kuo CH Camporesi E Bulgakov TS Lumyong S Jones EBG Chomnunti P Gentekaki E Bungartz F Zeng XY Fryar S Tkalčec Z Liang JM Li GS Wen TC Singh PN Gafforov Y Promputtha I Yasanthika E Goonasekara ID Zhao RL Zhao Q Kirk PM Liu JK Yan JY Mortimer PE Xu JC Doilom M (2019) Fungal diversity notes 1036–1150: Taxonomic and phylogenetic contributions on genera and species of fungal taxa. Fungal Diversity 96: 1–242. 10.1007/s13225-019-00429-2

[B15] Hyde KD Jeewon R Chen YJ Bhunjun CS Calabon MS Jiang HB Lin CG Norphanphoun C Sysouphanthong P Pem D Tibpromma S Zhang Q Doilom M Jayawardena RS Liu JK Maharachchikumbura SSN Phukhamsakda C Phookamsak R Al-Sadi AM Thongklang N Wang Y Gafforov Y Jones EBG Lumyong S (2020) The numbers of fungi: Is the descriptive curve flattening? Fungal Diversity 103: 219–271. 10.1007/s13225-020-00458-2

[B16] Hyde KD Suwannarach N Jayawardena RS Manawasinghe IS Liao CF Doilom M Cai L Zhao P Buyck B Phukhamsakda C Su WX Fu YP Li Y Zhao RL He MQ Li JX Tibpromma S Lu L Tang X Kang JC Ren GH Hofstetter V Ryoo R Antonín V Hurdeal VG Gentekaki E Zhang JY Lu YZ Senanayake IC Yu FM Zhao Q Bao DF (2021) Mycosphere notes 325–344–Novel species and records of fungal taxa from around the world. Mycosphere 12: 1101–1156. 10.5943/mycosphere/12/1/14

[B17] Hyde KD Norphanphoun C Ma J Yang HD Zhang JY Du TY Gao Y Gomes de Farias AR Gui H He SC He YK Li CJY Liu XF Lu L Su HL Tang X Tian XG Wang SY Wei DP Xu RF Xu RJ Yang Q Yang YY Zhang F Zhang Q Bahkali AH Boonmee S Chethana KWT Jayawardena RS Lu YZ Karunarathna SC Tibpromma S Wang Y Zhao Q (2023) Mycosphere notes 387–412–novel species of fungal taxa from around the world. Mycosphere 14(1): 663–744. 10.5943/mycosphere/14/1/8

[B18] Index Fungorum (2024) Index Fungorum. https://wwwindexfungorumorg/Names/Namesasp [Accessed 2 January 2024]

[B19] Jayasiri SC Hyde KD Ariyawansa HA Bhat J Buyck B Cai L Dai YC Abd-Elsalam KA Ertz D Hidayat I Jeewon R Jones EBG Bahkali AH Karunarathna SC Liu JK Luangsa-ard JJ Lumbsch HT Maharachchikumbura SSN McKenzie EHC Moncalvo JM Ghobad-Nejhad M Nilsson H Pang KL Pereira OL Phillips AJL Raspé O Rollins AW Romero AI Etayo J Selçuk F Stephenson SL Suetrong S Taylor JE Tsui CKM Vizzini A Abdel-Wahab MA Wen TC Boonmee S Dai DQ Daranagama DA Dissanayake AJ Ekanayaka AH Fryar SC Hongsanan S Jayawardena RS Li WJ Perera RH Phookamsak R de Silva NI Thambugala KM Tian Q Wijayawardene NN Zhao RL Zhao Q Kang JC Promputtha I (2015) The Faces of Fungi database: fungal names linked with morphology, phylogeny and human impacts. Fungal Diversity 74: 3–18. 10.1007/s13225-015-0351-8

[B20] Jayawardena RS Hyde KD Wang S Sun YR Suwannarach N Sysouphanthong P Abdel-Wahab MA Abdel-Aziz FA Abeywickrama PD Abreu VP Armand A Aptroot A Bao DF Begerow D Bellanger JM Bezerra JDP Bundhun D Calabon MS Cao T Cantillo T Carvalho JLVR Chaiwan N Chen CC Courtecuisse R Cui BK Damm U Denchev CM Denchev TT Deng CY Devadatha B de Silva NI dos Santos LA Dubey NK Dumez S Ferdinandez HS Firmino AL Gafforov Y Gajanayake AJ Gomdola D Gunaseelan S He SC Htet ZH Kaliyaperumal M Kemler M Kezo K Kularathnage ND Leonardi M Li JP Liao CF Liu S Loizides M Luangharn T Ma J Madrid H Mahadevakumar S Maharachchikumbura SSN Manamgoda DS Martín MP Mekala N Moreau PA Mu YH Pahoua P Pem D Pereira OL Phonrob W Phukhamsakda C Raza M Ren GC Rinaldi AC Rossi W Samarakoon BC Samarakoon MC Sarma VV Senanayake IC Singh A Souza MF Souza-Motta CM Spielmann AA Su WX Tang X Tian XG Thambugala KM Thongklang N Tennakoon DS Wannathes N Wei DP Welti S Wijesinghe SN Yang HD Yang YH Yuan HS Zhang H Zhang JY Balasuriya A Bhunjun CS Bulgakov TS Cai L Camporesi E Chomnunti P Deepika YS Doilom M Duan WJ Han SL Huanraluek N Jones EBG Lakshmidevi N Li Y Lumyong S Luo ZL Khuna S Kumla J Manawasinghe IS Mapook A Punyaboon W Tibpromma S Lu YZ Yan JY Wang Y (2022) Fungal diversity notes 1512–1610: Taxonomic and phylogenetic contributions on genera and species of fungal taxa. Fungal Diversity 117: 1–272. 10.1007/s13225-022-00513-0 36852303 PMC9948003

[B21] Jeewon R Hyde KD (2016) Establishing species boundaries and new taxa among fungi: Recommendations to resolve taxonomic ambiguities. Mycosphere 7(11): 1669–1677. 10.5943/mycosphere/7/11/4

[B22] Katoh K Standley DM (2013) Evolution MAFFT multiple sequence alignment software version 7: Improvements in performance and usability. Molecular Biology and Evolution 30(4): 772–780. 10.1093/molbev/mst010 23329690 PMC3603318

[B23] Konta S Tibpromma S Karunarathna SC Samarakoon MC Steven LS Mapook A Boonmee S Senwanna C Balasuriya A Eungwanichayapant PD Hyde KD (2023) Morphology and multigene phylogeny reveal ten novel taxa in Ascomycota from terrestrial palm substrates (Arecaceae) in Thailand. Mycosphere 14(1): 107–152. 10.5943/mycosphere/14/1/2

[B24] Li WL Liu ZP Zhang T Dissanayake AJ Luo ZL Su HY Liu JK (2021) Additions to *Distoseptispora* (Distoseptisporaceae) associated with submerged decaying wood in China. Phytotaxa 520: 75–86. 10.11646/phytotaxa.520.1.5

[B25] Liu YJ Whelen S Hall BD (1999) Phylogenetic relationships among ascomycetes: evidence from an RNA polymerse II subunit. Molecular Biology and Evolution 16: 1799–1808. 10.1093/oxfordjournals.molbev.a026092 10605121

[B26] Liu JW Hu YF Luo XX Xu ZH Castañeda-Ruíz RF Xia JW Zhang XG Zhang LH Cui RQ Ma J (2023) Morphological and phylogenetic analyses reveal three new species of *Distoseptispora* (Distoseptisporaceae, Distoseptisporales) from Yunnan, China. Journal of Fungi 9(4): 470. 10.3390/jof9040470 37108924 PMC10142134

[B27] Luo ZL Hyde KD Liu JK Bhat DJ Su H Bao DF Li WL (2018) Lignicolous freshwater fungi from China II: Novel *Distoseptispora* (Distoseptisporaceae) species from northwestern Yunnan Province and a suggested unified method for studying lignicolous freshwater fungi. Mycosphere 9: 444–461. 10.5943/mycosphere/9/3/2

[B28] Luo ZL Hyde KD Liu JK Maharachchikumbura SSN Jeewon R Bao DF Bhat DJ Lin CG Li WL Yang J Liu NG Lu YZ Jayawardena RS Li JF Su HY (2019) Freshwater Sordariomycetes. Fungal Diversity 99: 451–660. 10.1007/s13225-019-00438-1

[B29] Ma J Zhang JY Xiao XJ Xiao YP Tang X Boonmee S Kang JC Lu YZ (2022) Multi-gene phylogenetic analyses revealed five new species and two new records of Distoseptisporales from China. Journal of Fungi 8(11): е1202. 10.3390/jof8111202 PMC9697283 36422023

[B30] McKenzie EHC (1995) Dematiaceous hyphomycetes on Pandanaceae 5 *Sporidesmium**sensu lato*. Mycotaxon 56: 9–29.

[B31] Miller MA Pfeiffer W Schwartz T (2010) Creating the CIPRES Science Gateway for inference of large phylogenetic trees. Gateway Computing Environments Workshop (GCE), New Orleans (USA), November 2010, IEEE, 8 pp. 10.1109/GCE.2010.5676129

[B32] Monkai J Boonmee S Ren G Wei D Phookamsak R Mortimer PE (2020) *Distoseptisporahydei* sp. nov. (Distoseptisporaceae), a novel lignicolous fungus on decaying bamboo in Thailand. Phytotaxa 459: 93–107. 10.11646/phytotaxa.459.2.1

[B33] Nylander JAA (2004) MrModeltest 20 Program Distributed by the Author. Evolutionary Biology Centre, Uppsala University, Uppsala.

[B34] Phookamsak R Hyde KD Jeewon R Bhat DJ Jones EBG Maharachchikumbura SSN Raspé O Karunarathna SC Wanasinghe DN Hongsanan S Doilom M Tennakoon DS Machado AR Firmino AL Ghosh A Karunarathna A Mešić A Dutta AK Thongbai B Devadatha B Norphanphoun C Senwanna C Wei DP Pem D Ackah FK Wang GN Jiang HB Madrid H Lee HB Goonasekara ID Manawasinghe IS Kušan I Cano J Gené J Li JF Das K Acharya K Raj KNA Latha KPD Chethana KWT He MQ Dueñas M Jadan M Martín MP Samarakoon MC Dayarathne MC Raza M Park MS Telleria MT Chaiwan N Matočec N Silva NI Pereira OL Singh PN Manimohan P Uniya P Shang QJ Bhatt RP Perera RH Alvarenga RLM Nogal-Prata S Singh SK Vadthanarat S Oh SY Huang SK Rana S Konta S Paloi S Jayasiri SC Jeon SJ Mehmood T Gibertoni TB Nguyen TTT Singh U Thiyagaraja V Sarma VV Dong W Yu XD Lu YZ Lim YW Chen Y Tkalčec Z Zhang ZF Luo ZL Daranagama DA Thambugala KM Tibpromma S Camporesi E Bulgakov TS Dissanayake AJ Senanayake IC Dai DQ Tang LZ Khan S Zhang H Promputtha I Cai L Chomnunti P Zhao RL Lumyong S Boonmee S Wen TC Mortimer PE Xu JC (2019) Fungal diversity notes 929–1035: Taxonomic and phylogenetic contributions on genera and species of fungi. Fungal Diversity 95: 1–273. 10.1007/s13225-019-00421-w

[B35] Phukhamsakda C McKenzie EHC Phillips AJL Jones EBG Bhat DJ Marc S Bhunjun CS Wanasinghe DN Thongbai B Camporesi E Ertz D Jayawardena RS Perera RH Ekanayake AH Tibpromma S Doilom M Xu JC Hyde KD (2020) Microfungi associated with *Clematis* (Ranunculaceae) with an integrated approach to delimiting species boundaries. Fungal Diversity 102: 1–203. 10.1007/s13225-020-00448-4

[B36] Phukhamsakda C Nilsson RH Bhunjun CS Farias ARGD Sun YR Wijesinghe SN Raza M Bao DF Lu L Tibpromma S Dong W Tennakoon DS Tian XG Xiong YR Karunarathna SC Cai L Luo ZL Wang Y Manawasinghe IS Camporesi I Kirk PM Promputtha I Kuo CH Su HY Doilom M Li Y Fu YP Hyde KD (2022) The numbers of fungi: Contributions from traditional taxonomic studies and challenges of metabarcoding. Fungal Diversity 114: 327–386. 10.1007/s13225-022-00502-3

[B37] Rehner SA Samuels GJ (1994) Taxonomy and phylogeny of *Gliocladium* analysed from nuclear large subunit ribosomal DNA sequences. Mycological Research 98: 625–634. 10.1016/S0953-7562(09)80409-7

[B38] Ronquist F Teslenko M Van Der Mark P Ayres DL Darling A Höhna S Larget B Liu L Suchard MA Huelsenbeck JP (2012) MrBayes 32: Efficient Bayesian Phylogenetic Inference and model choice across a large model space. Systematic Biology 61: 539–542. 10.1093/sysbio/sys029 22357727 PMC3329765

[B39] Senanayake IC Rathnayaka AR Marasinghe DS Calabon MS Gentekaki E Lee HB Hurdeal VG Pem D Dissanayake LS Wijesinghe SN Bundhun D Goonasekara ID Abeywickrama PD Bhunjun CS Jayawardena RS Wanasinghe DN Jeewon R Bhat DJ Xiang MM (2020) Morphological approaches in studying fungi: collection, examination, isolation, sporulation and preservation. Mycosphere 11: 2678–2754. 10.5943/mycosphere/11/1/20

[B40] Shen HW Bao DF Hyde KD Su HY Bhat DJ Luo ZL (2021) Two novel species and two new records of *Distoseptispora* from freshwater habitats in China and Thailand. MycoKeys 84: 79–101. 10.3897/mycokeys.84.71905 34790026 PMC8592981

[B41] Shenoy BD Jeewon R Wu WP Bhat DJ Hyde KD (2006) Ribosomal and RPB2 DNA sequence analyses suggest that *Sporidesmium* and morphologically similar genera are polyphyletic. Mycological Research 110: 916–928. 10.1016/j.mycres.2006.06.004 16908125

[B42] Shoemaker RA White GP (1985) *Lasiosphaeriacaesariata* with *Sporidesmiumhormiscioides* and *L.triseptata* with *S.adscendens*. Sydowia 38: 278–283.

[B43] Song HY El Sheikha AF Zhai ZJ Zhou JP Chen MH Huo GH Huang XG Hu DM (2020) *Distoseptisporalongispora* sp. Nov. from freshwater habitats in China. Mycotaxon 135(3): 513–523. 10.5248/135.513

[B44] Stamatakis A Hoover P Rougemont J (2008) A rapid bootstrap algorithm for the RAxML web servers. Systematic Biology 57(5): 758–771. 10.1080/10635150802429642 18853362

[B45] Su HY Hyde KD Maharachchikumbura SSN Ariyawansa HA Luo ZL Promputtha I Tian Q Lin CG Shang QJ Zhao YC Chai HM Liu XY Bahkali AH Bhat JD McKenzie EHC Zhou DQ (2016) The families Distoseptisporaceae fam. Nov., Kirschsteiniotheliaceae, Sporormiaceae, and Torulaceae, with new species from freshwater in Yunnan Province, China. Fungal Diversity 80: 375–409. 10.1007/s13225-016-0362-0

[B46] Sun Y Goonasekara ID Thambugala KM Jayawardena RS Wang Y Hyde KD (2020) *Distoseptisporabambusae* sp. nov. (Distoseptisporaceae) on bamboo from China and Thailand. Biodiversity Data Journal 8: e53678. 10.3897/BDJ.8.e53678.figure2 PMC7280319 32547305

[B47] Swofford DL (2002) PAUP*: Phylogenetic analysis using parsimony (and other methods), version 40 b10 MA: Sinauer Associates, Sunderland.

[B48] Tibpromma S Hyde KD McKenzie EHC Bhat DJ Phillips AJL Wanasinghe DN Samarakoon MC Jayawardena RS (2018) Fungal diversity notes 840–928: Micro-fungi associated with Pandanaceae. Fungal Diversity 100: 1–160. 10.1007/s13225-018-0408-6

[B49] Vaidya G Lohman DJ Meier R (2011) SequenceMatrix: Concatenation software for the fast assembly of multi-gene datasets with character set and codon information. Cladistics 27: 171–180. 10.1111/j.1096-0031.2010.00329.x 34875773

[B50] Vilgalys R Hester M (1990) Rapid genetic identification and mapping of enzymatically amplified ribosomal DNA from several *Cryptococcus* species. Journal of Bacteriology 172: 4238–4246. 10.1128/jb.172.8.4238-4246.1990 2376561 PMC213247

[B51] White TJ Bruns T Lee SJWT Taylor J (1990) Amplification and direct sequencing of fungal ribosomal RNA genes for phylogenetics. PCR protocols: A guide to methods and applications 18(1): 315–322. 10.1016/B978-0-12-372180-8.50042-1

[B52] Wu WP Zhuang WY (2005) *Sporidesmium*, *Endophragmiella* and related genera from China. Fungal Diversity Research Series 15: 1–351.

[B53] Xia JW Ma YR Li Z Zhang XG (2017) Acrodictys-like wood decay fungi from southern China, with two new families Acrodictyaceae and Junewangiaceae. Scientific Reports 7: e7888. 10.1038/s41598-017-08318-x PMC5554248 28801663

[B54] Yang J Maharachchikumbura SSN Liu JK Hyde KD Jones EBG Al-Sadi AM Liu ZY (2018) *Pseudostanjehughesiaaquitropica* gen. et sp. nov. and *Sporidesmium**sensu lato* species from freshwater habitats. Mycological Progress 17: 591–616. 10.1007/s11557-017-1339-4

[B55] Yang J Liu LL Jones EBG Li WL Hyde KD Liu ZY (2021) Morphological variety in *Distoseptispora* and introduction of six novel species. Journal of Fungi 7: e945. 10.3390/jof7110945 PMC8620209 34829232

[B56] Zhai ZJ Yan JQ Li WW Gao Y Hu HJ Zhou JP Song HY Hu DM (2022) Three novel species of *Distoseptispora* (Distoseptisporaceae) isolated from bamboo in Jiangxi Province, China. MycoKeys 88: 35–54. 10.3897/mycokeys.88.79346 36478919 PMC9633981

[B57] Zhang H Zhu R Qing Y Yang H Li CX Wang GN Zhang D Ning P (2022) Polyphasic identification of *Distoseptispora* with six new species from freshwater. Journal of Fungi 8: e1063. 10.3390/jof8101063 PMC9605234 36294625

